# Influence of *CYP3A4/*5 and *ABC* transporter polymorphisms on lenvatinib plasma trough concentrations in Japanese patients with thyroid cancer

**DOI:** 10.1038/s41598-019-41820-y

**Published:** 2019-04-01

**Authors:** Tomoko Ozeki, Mitsuji Nagahama, Kazuma Fujita, Akifumi Suzuki, Kiminori Sugino, Koichi Ito, Masatomo Miura

**Affiliations:** 1 Department of Pharmacy, Akita University Hospital, Akita, Japan; 2 Department of Surgery, Ito Hospital, Tokyo, Japan

## Abstract

Lenvatinib is a substrate of cytochrome P450 (CYP) 3A and ATP-binding cassette (ABC) transporters. In this study, we aimed to evaluate how *CYP3A4/5* and *ABC* transporter polymorphisms affected the mean steady-state dose-adjusted plasma trough concentrations (C_0_) of lenvatinib in a cohort of 40 Japanese patients with thyroid cancer. *CYP3A4* 20230G > A (*1G), *CYP3A5* 6986A > G (*3), *ABCB1* 1236C > T, *ABCB1* 2677G > T/A, *ABCB1* 3435C > T, *ABCC2* −24C > T, and *ABCG2* 421C > A genotypes were determined using polymerase chain reaction-restriction fragment length polymorphism. In univariate analysis, there were no significant differences in the mean dose-adjusted C_0_ values of lenvatinib between the *ABCB1*, *ABCG2*, and *CYP3A5* genotypes. However, the mean dose-adjusted C_0_ values of lenvatinib in patients with the CYP3A4*1/*1 genotype and *ABCC2* −24T allele were significantly higher than those in patients with the CYP3A4*1G allele and −24C/C genotype, respectively (*P* = 0.018 and 0.036, respectively). In multivariate analysis, *CYP3A4* genotype and total bilirubin were independent factors influencing the dose-adjusted C_0_ of lenvatinib (*P* = 0.010 and 0.046, respectively). No significant differences were found in the incidence rates of hypertension, proteinuria, and hand-foot syndrome following treatment with lenvatinib between the genotypes of *CYP3A4/5* and *ABC* transporters. Lenvatinib pharmacokinetics were significantly influenced by the CYP3A4*1G polymorphism. If the target plasma concentration of lenvatinib for efficacy or toxicity is determined, elucidation of the details of the CYP3A4*1G genotype may facilitate decision-making related to the appropriate initial lenvatinib dosage to achieve optimal plasma concentrations.

## Introduction

Lenvatinib is an oral tyrosine kinase inhibitor (TKI) that inhibits vascular endothelial growth factor receptor (VEGFR) 1–3, fibroblast growth factor receptor (FGFR) 1–4, platelet-derived growth factor receptor α (PDGFRα), stem cell factor receptor (KIT), and rearranged during transfection (RET)^1–4^.

Lenvatinib is primarily metabolized by cytochrome P450 (CYP) 3A4 and acts as a substrate for ATP-binding cassette (ABC) transporters, such as P-glycoprotein and breast cancer resistance protein (BCRP), which are encoded by the *ABCB1* and *ABCG2* genes, respectively^5–8^. P-glycoprotein and BCRP are expressed in the small intestine, liver, kidney, and blood-brain barrier and are associated with drug disposition, functioning to regulate the absorption and elimination of substrate drugs^9^. Co-administration of the potent CYP3A, P-glycoprotein, and BCRP inhibitor ketoconazole has been reported to increase the maximum plasma concentration of lenvatinib compared with placebo; however, the elimination half-life of lenvatinib was not altered^5^. This result suggested that CYP3A, P-glycoprotein, and BCRP in the small intestine contribute to the pharmacokinetics of lenvatinib. Therefore, the pharmacokinetics of lenvatinib could be affected by polymorphisms in the *CYP3A4*, *ABCB1*, and *ABCG2* genes. Furthermore, CYP3A4 and CYP3A5 generally have similar catalytic specificities, although the activity of CYP3A5 is less than that of CYP3A4^10,11^. Polymorphic CYP3A5 expression in the small intestine and liver is strongly correlated with a single nucleotide polymorphism (SNP), 6986A > G, within intron 3 of *CYP3A5*, which is designated CYP3A5*3^12^. Therefore, lenvatinib pharmacokinetics may be influenced by *CYP3A5* polymorphisms. However, the effects of *CYP3A4*/*5*, *ABCB1*, and *ABCG2* polymorphisms on the plasma concentrations of lenvatinib have not been evaluated to date.

In addition, in humans, three ABC subfamilies, B, C, and G, contain efflux transporters for a large number of drugs. Among these ABC subfamilies, *ABCB1*, *ABCG2*, and *ABCC2* (multidrug resistance-associated protein [MRP] 2) are important for the drug transporters. To date, however, in both *in vitro* and *in vivo* studies, the influence of MRP2 on membrane transport of lenvatinib has not been reported. In addition to P-glycoprotein and BCRP, MRP2 may also play a role in lenvatinib pharmacokinetics. In addition, no studies have reported the effects of *ABCC2* polymorphisms on lenvatinib plasma concentrations. Therefore, in the present study, we investigated the effects of polymorphisms in *CYP3A4* 20230G > A, *CYP3A5* 6986A > G, *ABCB1* 1236C > T, *ABCB1* 2677G > T/A, *ABCB1* 3435C > T, *ABCG2* 421C > A, and *ABCC2* −24C > T on the mean steady-state dose-adjusted plasma trough concentration (C_0_) of lenvatinib in 40 patients with thyroid cancer.

## Methods

### Patients and protocols

Forty Japanese patients with thyroid cancer (27 women and 13 men) taking lenvatinib (LENVIMA; Eisai Co., Ltd., Tokyo, Japan) were consecutively enrolled in this study. All patients were treated at Ito Hospital between June 2015 and April 2018. The eligibility criteria were as follows: adequate bone marrow function (neutrophil count of 1500/μL or more, platelet count of 100,000/μL or more, and haemoglobin level of 8.0 g/dL or more), renal function (serum creatinine levels less than 2.0 mg/dL), and hepatic function (total bilirubin level less than 2.0 mg/dL, aspartate transferase and alanine transferase levels less than or equal to 2.5 times the upper limit of the normal range). Table 1 lists the clinical characteristics of patients prior to lenvatinib therapy. Patients were excluded if they had taken drugs that affected CYP3A, P-glycoprotein, or BCRP function. Lenvatinib was administered as medical care for patients with thyroid cancer according to a Drug Interview Form^8^. The study was approved by the Ethics Committee of Ito Hospital and Akita University School of Medicine and was carried out according to the recommendations outlined in the 1964 Declaration of Helsinki and its later amendments. All patients who participated in this study provided written informed consent prior to enrolment.

**Table 1:** Clinical characteristics of patients receiving lenvatinib.

Characteristics	
Total number	40
Female:Male	27:13
Age (years)	63.0 ± 10.9 (33–78)
Body weight (kg)	56.0 ± 11.0 (35–82)
Laboratory test values at lenvatinib initiation	
Aspartate transaminase (IU/L)	25.9 ± 8.9 (13–56)
Alanine transaminase (IU/L)	22.3 ± 9.1 (11–45)
Serum albumin (g/dL)	3.9 ± 0.5 (2.7–5.1)
Total bilirubin (mg/dL)	0.6 ± 0.2 (0.4–1.5)
Serum creatinine (mg/dL)	0.76 ± 0.29 (0.41–1.78)
*ABCB1* 1236C > T	C/C: C/T: T/T = 8: 22: 10
*ABCB1* 2677G > T/A	G/G: G/T: G/A: T/T: T/A: A/A = 9: 15: 4: 4: 5: 3
*ABCB1* 3435C > T	C/C: C/T: T/T = 14: 21: 5
*ABCG2* 421C > A	C/C: C/A: A/A = 20: 18: 2
*ABCC2* −24C > T	C/C: C/T: T/T = 24: 14: 2
CYP3A4 20230 G > A(*1G)	*1/*1: *1/*1G: *1G/*1G = 24: 15: 1
*CYP3A5* 6986 A > G(*3)	*1/*1: *1/*3: *3/*3 = 1: 13: 26

Data are presented as number or mean ± standard deviation (range).

**Figure 1:**
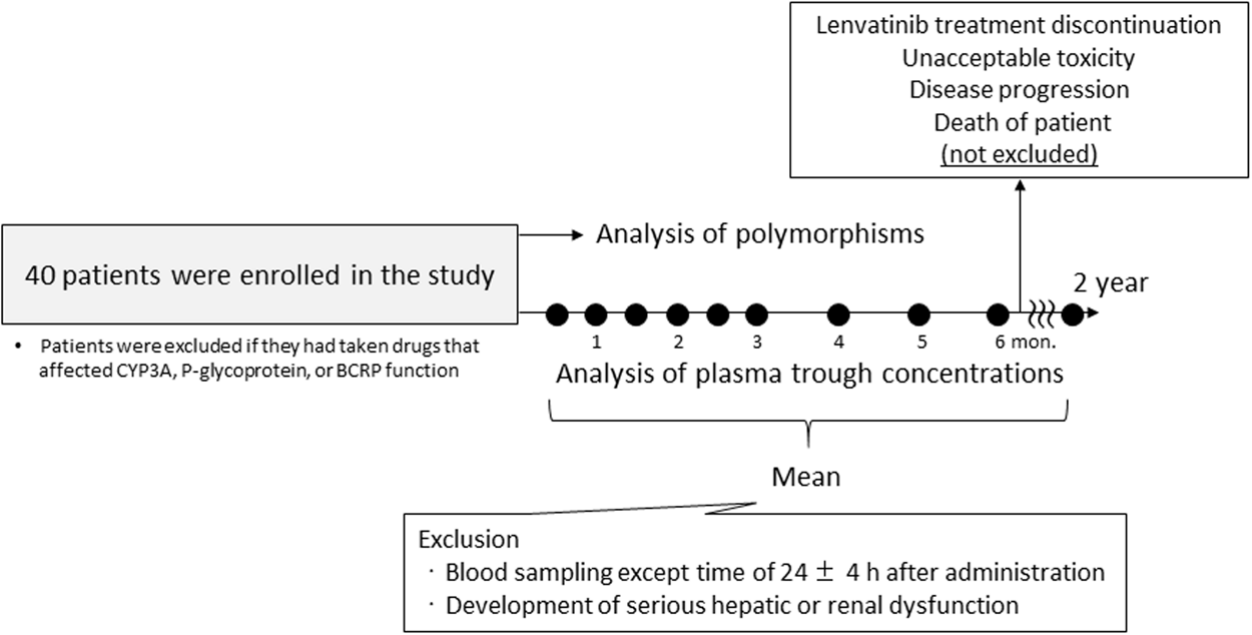
Study profile.

Oral lenvatinib administration was carried out at dosages of 4–24 mg once daily. The daily dose of lenvatinib was reduced as needed based on the grade of each side effect, as determined by the Common Terminology Criteria for Adverse Events version 4.0. Intake of foods or drugs known to alter CYP3A, P-glycoprotein, or BCRP function was not permitted during the treatment period (withdrawal criteria). The plasma concentrations of lenvatinib were monitored 24 ± 4 h after administration, and trough plasma concentrations (C_0_) were evaluated every 2 weeks for the first 3 months after initiation of lenvatinib therapy and then every 1 month thereafter. Assays of lenvatinib C_0_ were performed monthly until lenvatinib treatment discontinuation (unacceptable toxicity, disease progression, or death of patient) or for a maximum of 2 years (maximum 27 sampling points). Blood samples except the sampling time of 24 ± 4 h after administration and the steady-state was excluded from analysis. In addition, plasma samples from patients who developed serious hepatic or renal dysfunction after initiation of lenvatinib therapy were excluded from analysis because of the influence on lenvatinib pharmacokinetics (Fig. 1). Plasma was isolated by centrifugation at 1,900 × *g* for 15 min and was stored at −40 °C until analysis.

### Pharmacokinetics analysis

Plasma concentrations of lenvatinib were measured by high-performance liquid chromatography (HPLC), as has been used previously for quantitative analysis of gefitinib plasma concentrations^13^. Analysis of the calibration curve for lenvatinib in human plasma showed that the curve was linear for the concentration range from 5 to 1000 ng/mL. Moreover, for this assay, the limit of quantification (LOQ) of lenvatinib was 5 ng/mL. For the concentration range from 5 to 1000 ng/mL, the coefficients of variation (CVs) were lower than 12.6%, and the accuracies of the intra- and interday assays were within 10.6%.

### Pharmacogenomics analysis

Extraction of DNA from peripheral blood samples was carried out using a QIAamp Blood Mini Kit (Qiagen, Tokyo, Japan). Genotypes *CYP3A4* 20230G > A (*1G) (rs2242480), within intron 10 of the human *CYP3A4* gene, and *CYP3A5* 6986A > G (*3) (rs776746) were identified using polymerase chain reaction-restriction fragment length polymorphism (PCR-RFLP)^14^. Genotyping procedures identifying the C and T alleles in exon 12 (1236C > T, rs1128503), the G and T/A alleles in exon 21 (2677G > T/A, rs2032582), and the C and T alleles in exon 26 (3435C > T, rs1045642) of the *ABCB1* gene were performed using PCR-RFLP as previously reported^15–17^. PCR-RFLP was used for genotyping of the *ABCG2* 421C > A (rs2231142) polymorphism, as described by Kobayashi *et al*.^18^. Genotyping procedures identifying the C and T alleles in exon 1 (−24C > T, rs717620) of the *ABCC2* gene were carried out using PCR-RFLP, as described by Naesens *et al*.^19^. All frequencies for the different analysed loci were at Hardy-Weinberg equilibrium.

### Statistical analyses

The Kolmogorov-Smirnov test was applied to assess the distribution in each data set. The results used the average of dose-adjusted C_0_ values of lenvatinib during therapy for one patient. Dose-adjusted C_0_ values for each genotype were presented as medians (minimum–maximum). To elucidate differences between these groups, we applied Kruskal-Wallis tests or Mann-Whitney U tests. Correlations in continuous variables between groups were determined using Spearman’s rank correlation coefficient test, with results presented as correlation coefficients (*r*). The effects of factors in univariate analysis were evaluated using stepwise multiple linear regression analysis. For each patient, dummy variables were used to replace the genotypes of *CYP3A4/5* and *ABC* transporters (1 and 0 in 2 groups; 1 and 0, 0 and 0, and 0 and 1 in 3 groups). Chi-square tests were used to examine differences in categorical data. For post-hoc power analysis, the effect size calculated in comparison with the lenvatinib dose-adjusted C_0_ between *CYP3A4* or *ABCC2* genotypes showed significant differences. An effect size of greater than 0.5 was considered clinically meaningful^20^. Power was calculated using G*Power ver. 3.1 software. Results with *p* values of less than 0.05 were considered significant, and SPSS 20.0 for Windows (SPSS IBM Japan Inc., Tokyo, Japan) was used for all statistical analyses.

## Results

The genotype frequencies for the polymorphisms in *ABC* transporters and *CYP3A4/5* in 40 Japanese patients with thyroid cancer are shown in Table 1. The median (range) CV values of the lenvatinib C_0_ at the steady state for one patient (intrapatient) for the same daily dose during lenvatinib therapy were 20.8% (8.9–54.4%) at 24 mg, 15.3% (5.7–63.8%) at 20 mg, 25.6% (3.4–66.2%) at 14 mg, 24.2% (4.8–72.9%) at 10 mg, 32.8% (16.3–50.9%) at 8 mg, and 23.5% (7.1–39.8%) at 4 mg. Therefore, the mean lenvatinib plasma concentration during lenvatinib therapy was used. The median (range) duration of lenvatinib therapy was 394 (64–735) days.

**Figure 2:**
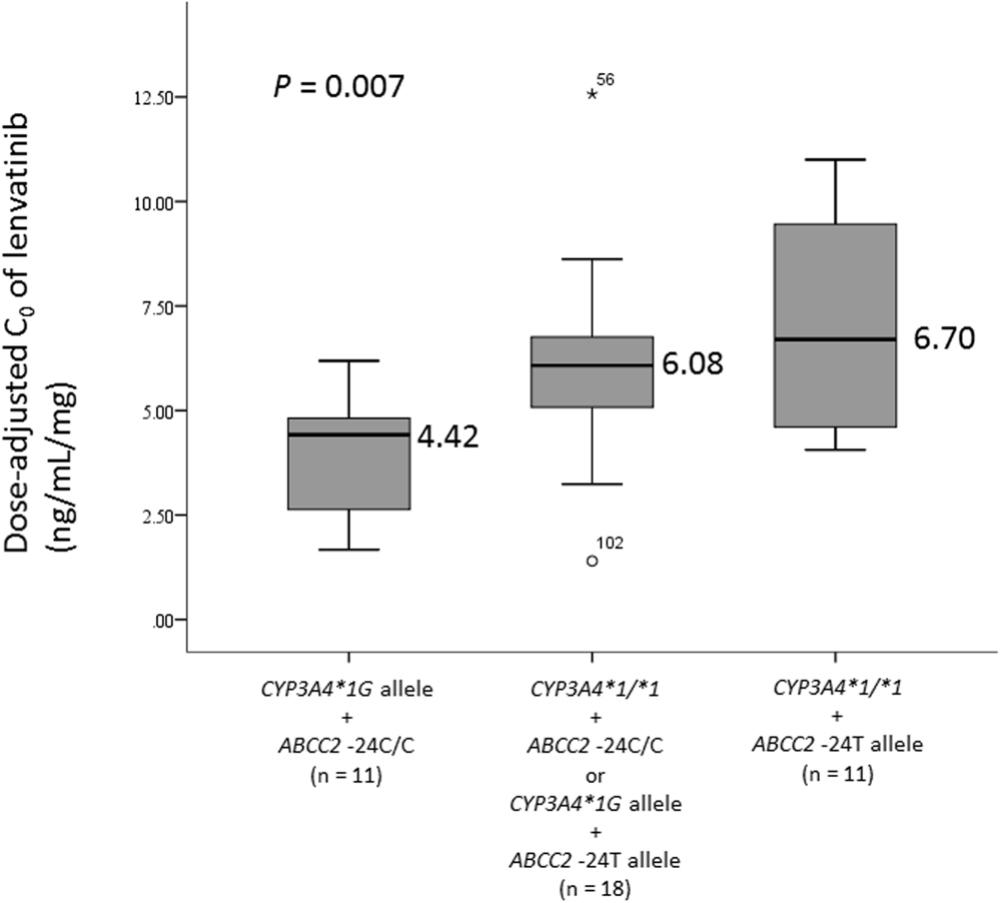
Effects of CYP3A4*1G and *ABCC2* −24C > T polymorphisms on the steady-state mean plasma trough concentrations of lenvatinib. Graphical analysis was performed using a box and whiskers plot. The box spanned data between 2 quartiles (IQR), with the median represented as a bold horizontal line. The ends of the whiskers (vertical lines) represent the smallest and largest values that are not outliers. Outliers (circle) are values between 1.5 and 3 IQRs from the end of the box. Values more than three IQRs from the end of the box are defined as extremes (stars).

There were no significant differences in the mean dose-adjusted C_0_ of lenvatinib between genotypes for the *ABCB1*, *ABCG2*, and *CYP3A5* polymorphisms, although the dose-adjusted C_0_ of lenvatinib in patients with the *ABCG2* 421A allele or CYP3A5*3/*3 tended to be higher than those with other corresponding genotypes (Table 2). However, the mean dose-adjusted C_0_ of lenvatinib in patients with the CYP3A4*1/*1 genotype was significantly higher than that in patients with the CYP3A4*1G allele (*P* = 0.018, effect size = 0.863; Table 2). In addition, the mean dose-adjusted C_0_ of lenvatinib in patients with the *ABCC2* −24T allele was significantly higher than that in patients with −24C/C genotypes (*P* = 0.036, effect size = 0.605; Table 2). There were also significant correlations with total bilirubin at lenvatinib initiation and the mean dose-adjusted C_0_ of lenvatinib at the steady state (*P* = 0.037; Table 2). The dose-adjusted C_0_ of lenvatinib in patients with both the CYP3A4*1/*1 and *ABCC2* −24T alleles (median 6.70 ng/mL/mg) was about 1.5-fold higher than that in patients with both the CYP3A4*1G/*1G and *ABCC2* −24C/C genotypes (median 4.42 ng/mL/mg; *P* = 0.007; Fig. 2).

**Table 2:** Comparison of dose-adjusted mean trough concentrations of lenvatinib between genotypes of *ABC* transporters and *CYP3A4/5*.

Genotype	n	Dose-adjusted C_0_ (ng/mL/mg)		*P* value
		Median	Minimum - Maximum	
*ABCB1* 1236C > T				
C/C	8	4.37	(1.67–10.4)	0.303^a^
C/T	22	5.87	(1.40–12.6)	
T/T	10	5.85	(3.24–11.0)	
*ABCB1* 2677G > T/A				
G/G	9	5.08	(3.09–12.6)	0.755^a^
G/T + G/A	19	5.67	(1.40–11.0)	
T/T + T/A + A/A	12	5.62	(2.06–11.0)	
*ABCB1* 3435C > T				
C/C	14	4.90	(1.40–12.6)	0.724^a^
C/T	21	5.52	(2.06–11.0)	
T/T	5	6.02	(4.24–7.46)	
*ABCG2* 421C > A				
C/C	20	4.73	(1.40–12.6)	0.063^b^
C/A + A/A	20	6.34	(1.67–10.4)	
*ABCC2* −24C > T				
C/C	24	5.03	(1.40–12.6)	0.036^b^
C/T + T/T	16	6.57	(4.06–11.0)	
*CYP3A4* 20230G > A(*1G)				
*1/*1	24	6.34	(1.40–12.6)	0.018^b^
*1/*1 *G* + *1 G/*1G	16	4.77	(1.67–7.52)	
*CYP3A5* 6986 A > G(*3)				
*1/*1 + *1/*3	14	4.77	(1.67–10.4)	0.063^b^
*3/*3	26	6.22	(1.40–12.6)	
Sex				
Female	27	5.67	(1.67–12.6)	0.252^b^
Male	13	5.08	(1.40–10.4)	
	Correlation coefficient (*r*)			
Age (years)	0.061			0.817
Body weight (kg)	−0.220			0.414
Laboratory test values at lenvatinib initiation				
Aspartate transaminase	0.273			0.098
Alanine transaminase	0.151			0.388
Serum albumin	0.189			0.249
Total bilirubin	0.336			0.037
Serum creatinine	−0.151			0.357

^a^Kruskal Wallis, ^b^Mann-Whitney.

The dose-adjusted C_0_ of lenvatinib for each patient was calculated using the average of several C_0_ values at the steady-state.

Stepwise selection multiple linear regression analysis of explanatory variables for the dose-adjusted C_0_ values of lenvatinib is shown in Table 3. The *CYP3A4* genotype (CYP3A4*1/*1) and total bilirubin were independent factors influencing the dose-adjusted C_0_ of lenvatinib (*P* = 0.010 and 0.046, respectively); however, the determination coefficient for the dose-adjusted C_0_ of lenvatinib was 0.226.

**Table 3:** Stepwise multiple regression analysis of explanatory variables for the dose-adjusted C_0_ of lenvatinibTable 3. Stepwise multiple regression analysis of explanatory variables for the dose-adjusted C_0_ of lenvatinib.

Explanatory variable	Slope	SE	SRC	*P*- value	*R* ^2^
					0.226
CYP3A4 genotype (*1G allele = 1)	−2.05	0.75	−0.397	0.010	
Total bilirubin (mg/dL)	3.67	1.77	0.300	0.046	
Intercept =	6.39	1.66			

C_0_, predose concentration.

SE, standard error; SRC, standardized regression coefficient.

No significant differences were found in the incidence rates of hypertension, proteinuria, and hand-foot syndrome following treatment with lenvatinib between the genotypes of *CYP3A4/5* and *ABC* transporters (Table 4).

**Table 4:** The frequencies of lenvatinib-induced adverse events between genotypes of *ABC* transporters and *CYP3A4/5*.

Adverse events	Hypertension				*P* value	Proteinuria				*P* value	Hand-foot syndrome			*P* value
CTCAE grade	0	1	2	3		0	1	2	3		0	1	2	
***ABCB1*** **1236C>T**														
C/C	0	4	3	1	0.140	3	2	3	0	0.599	0	6	2	0.431
C/T	0	3	16	3		11	3	6	2		0	14	8	
T/T	0	1	6	3		6	3	1	0		1	7	2	
***ABCB1*** **2677G>T/A**														
G/G	0	0	8	1	0.211	3	2	4	0	0.387	0	7	2	0.702
G/T + G/A	0	4	10	5		11	4	2	2		1	11	7	
T/T + T/A + A/A	0	4	7	1		6	2	4	0		0	9	3	
***ABCB1*** **3435C>T**														
C/C	0	3	10	1	0.798	4	3	7	0	0.093	0	13	1	0.150
C/T	0	4	12	5		13	3	3	2		1	11	9	
T/T	0	1	3	1		3	2	0	0		0	3	2	
***ABCG2*** **421C>A**														
C/C	0	5	11	4	0.606	11	4	4	1	0.896	0	14	6	0.595
C/A + A/A	0	3	14	3		9	4	6	1		1	13	6	
***ABCC2*** **−24C>T**														
C/C	0	3	17	4	0.305	11	5	7	1	0.866	1	15	8	0.574
C/T + T/T	0	5	8	3		9	3	3	1		0	12	4	
***CYP3A4*** **20230G>A(** *1G **)**														
*1/*1	0	6	14	4	0.625	14	3	6	1	0.457	1	14	9	0.281
*1/*1G + *1G/*1G	0	2	11	3		6	5	4	1		0	13	3	
***CYP3A5*** 6986A>G(*3)														
*1/*1 + *1/*3	0	3	9	2	0.923	5	4	4	1	0.577	0	12	2	0.185
*3/*3	0	5	16	5		15	4	6	1		1	15	10	

Data are presented as patients numbers.

## Discussion

To the best of our knowledge, this is the first study to report the effects of *CYP3A4/5* and *ABC* transporter polymorphisms on lenvatinib pharmacokinetics in patients with thyroid cancer. Our findings showed that lenvatinib pharmacokinetics were significantly influenced by the *CYP3A4* 20230G > A polymorphism; however, the *ABCB1*, *ABCG2*, and *CYP3A5* polymorphisms had no effect. Notably, univariate analysis showed that the dose-adjusted C_0_ of lenvatinib was significantly higher in patients having the *ABCC2* −24T allele than in patients having the −24C/C genotype (*P* = 0.036). Accordingly, in patients with both the CYP3A4*1G/*1G and *ABCC2* −24C/C genotypes, the dose-adjusted C_0_ of lenvatinib was significantly lower than that in patients with other genotypes. However, the appearances of hypertension, proteinuria, and hand-foot syndrome following lenvatinib treatment appeared to be unrelated to these genetic polymorphisms.

Eighty-eight percent of cases of the CYP3A4*1G polymorphism 20230G > A have been reported to be linked to CYP3A5*1^21^. Notably, in a previous study of patients harboring the CYP3A4*1G allele, metabolic activity for the substrate of CYP3A4 was increased; that is, patients harboring the CYP3A4*1G allele showed significantly lower dose-adjusted C_0_ values of tacrolimus than patients harboring the CYP3A4*1/*1 genotype^14^. As previously demonstrated^21^, patients with the CYP3A4*1G allele exhibited significantly lower dose-adjusted C_0_ values for lenvatinib than patients having the CYP3A4*1/*1 genotype. However, the dose-adjusted C_0_ values of lenvatinib did not differ significantly between the 2 *CYP3A5* genotypes. Twelve percent of CYP3A4*1G polymorphisms that are not linked to CYP3A5*1 seem to show increased correlations between CYP3A4*1G polymorphisms and lenvatinib pharmacokinetics.

In an *in vitro* study, the *ABCC2* −24T variant was reported to be associated with decreased transport activity of MRP2^22^. Similarly, in the present study, the dose-adjusted C_0_ of lenvatinib was also significantly increased in patients having the *ABCC2* −24T allele compared with that in patients having the −24C/C genotype. Therefore, *ABCC2* polymorphism may also contribute to interindividual differences in lenvatinib pharmacokinetics. However, the SNP in position 421C > A of *ABCG2* is the most prevalent allele in Japanese individuals (33%) and exhibits functional importance^23^. The level and function of ABCG2 protein expressed from the 421A allele are reduced compared with those of the 421C/C protein^18^. To date, the plasma concentrations of other TKIs, particularly imatinib, in patients with the *ABCG2* 421A allele have been reported to be higher than those in patients with the 421C/C genotype^24,25^. Notably, our current findings demonstrated that in patients having the *ABCG2* 421A allele, the dose-adjusted C_0_ of lenvatinib tended to be higher than in patients having the 421C/C genotype, although this difference was not significant. Furthermore, each polymorphism in the human *ABCB1* gene (1236C > T, 2677G > T/A, and 3435C > T) has been associated with altered expression and function of P-glycoprotein in various human tissues^15–17^. The dose-adjusted C_0_ of lenvatinib in patients with the *ABCB1* 1236 T/T or 3435 T/T genotype also tended to be higher than that in patients with the corresponding 1236C/C or 3435C/C genotype. Accordingly, further studies with larger sample sizes are needed; however, in addition to P-glycoprotein and BCRP, MRP2 may also contribute to the absorption and disposition of lenvatinib. MRP2 is localized at the apical membrane of enterocytes in the intestine and plays a key role in limiting the absorption of substrate drugs incorporated orally. In the liver, MRP2 localizes to the canalicular membrane of hepatocytes and mediates the efflux of substrate drugs into the bile^26^.

Our current results showed that patients having both the CYP3A4*1/*1 and *ABCC2* −24T alleles administered the same daily dose of lenvatinib exhibited lenvatinib C_0_ values about 1.5-fold higher than those in patients having both the CYP3A4*1G/*1G and *ABCC2* −24C/C genotypes. Higher lenvatinib plasma concentrations may be associated with higher rates of some adverse events. In lenvatinib therapy, evaluation of lenvatinib plasma concentrations may be necessary to provide individual treatment through dose adjustment because of improved efficacy or to avoid adverse events. Identification of therapeutic target plasma concentrations indicating the relationships of drug exposure-efficacy or toxicity will then be required. When target plasma concentrations of lenvatinib are elucidated, the present results may be useful clinically. Specifically, elucidation of information regarding the primary *CYP3A4* genotype and minor *ABCC2* genotype prior to initiation of lenvatinib therapy may facilitate decision-making related to the appropriate daily dosage for obtaining optimal lenvatinib plasma exposure. Further studies are necessary in the future to examine this possibility.

Our study had several limitations. First, the number of patients treated with lenvatinib therapy was only 40, which may have hindered the precise determination of lenvatinib pharmacogenomics. Second, because some polymorphisms were of low frequency, the associations observed may have been accidental. Therefore, further studies with larger patient cohorts are needed to confirm our results. However, we investigated the validity of the sample size from the results obtained from the study and found that the effect sizes in comparison with the lenvatinib dose-adjusted C_0_ values between *CYP3A4* or *ABCC2* genotypes were 0.863 and 0.605, respectively, supporting the clinical significance of the results.

## Conclusion

Overall, our results demonstrated that the mean steady-state dose-adjusted C_0_ of lenvatinib was significantly affected by *CYP3A4* polymorphism but not *ABCB1*, *ABCG2*, or *CYP3A5* polymorphisms. In addition to P-glycoprotein and BCRP, MRP2 may also contribute to the absorption and disposition of lenvatinib. If the target plasma concentration of lenvatinib for efficacy or toxicity is determined, elucidation of details of the CYP3A4*1G genotype may facilitate decision-making related to the appropriate initial lenvatinib dosage to achieve optimal plasma concentrations.
